# HMGB1 regulates ferroptosis through Nrf2 pathway in mesangial cells in response to high glucose

**DOI:** 10.1042/BSR20202924

**Published:** 2021-02-19

**Authors:** You Wu, Ying Zhao, Han-ze Yang, Yan-jun Wang, Yan Chen

**Affiliations:** Department of Endocrinology, The Second Hospital of Jilin University, Changchun 130041, China

## Abstract

Ferroptosis, a novel type of programmed cell death, is involved in inflammation and oxidation of various human diseases, including diabetic kidney disease. The present study explored the role of high-mobility group box-1 (HMGB1) on the regulation of ferroptosis in mesangial cells in response to high glucose. Compared with healthy control, levels of serum ferritin, lactate dehydrogenase (LDH), reactive oxygen species (ROS), malonaldehyde (MDA), and HMGB1 were significantly elevated in diabetic nephropathy (DN) patients, accompanied with deregulated ferroptosis-related molecules, including long-chain acyl-CoA synthetase 4 (ACSL4), prostaglandin-endoperoxide synthase 2 (PTGS2), NADPH oxidase 1 (NOX1), and glutathione peroxidase 4 (GPX4). *In vitro* assay revealed that erastin and high glucose both induced ferroptosis in mesangial cells. Suppression of HMGB1 restored cellular proliferation, prevented ROS and LDH generation, decreased ACSL4, PTGS2, and NOX1, and increased GPX4 levels in mesangial cells. Furthermore, nuclear factor E2-related factor 2 (Nrf2) was decreased in DN patients and high glucose-mediated translocation of HMGB1 in mesangial cells. Knockdown of HMGB1 suppressed high glucose-induced activation of TLR4/NF-κB axis and promoted Nrf2 expression as well as its downstream targets including HO-1, NQO-1, GCLC, and GCLM. Collectively, these findings suggest that HMGB1 regulates glucose-induced ferroptosis via Nrf2 pathway in mesangial cells.

## Introduction

Diabetic kidney disease is one of the most common complications of diabetes characterized by macroalbuminuria, hypertension, and decreased glomerular filtration rate (GFR) [1]. It leads the cause of end-stage renal disease (ESRD) in developed countries and accounts for approximately 16.4% of ESRD cases in China, and accounts for a significant increase in morbidity and mortality in patients with diabetes [2]. Thus, it is critical to elucidate the pathogenesis and develop therapeutic interventions of diabetic kidney disease.

Programmed cell death is regulated by an intricate mechanism that is closely related to inflammatory processes triggered by innate immune responses. Ferroptosis, a novel type of programmed cell death, has been proved to be involved in inflammation and oxidation of various human diseases [3]. Ferroptosis is morphologically, biochemically and genetically distinct from apoptosis, necrosis, and autophagy [3,4]. It is characterized by the iron-dependent accumulation of reactive oxygen species (ROS) and lipid peroxidation, which may be inhibited by lipophilic antioxidants, iron chelators, and inhibitors of lipid peroxidation [3]. Although ferroptosis is implicated in human diseases, its precise regulatory mechanism and biological functions remain elusive.

High-mobility group box-1 (HMGB1) is a transcription factor that is involved in chromatin remodeling and DNA recombination and repair processes. It is abundant in the cell nucleus and is significantly expressed in alveolar epithelial cells, alveolar macrophages, and infiltrating inflammatory cells [5]. HMGB1 inside the cytosol is translocated to be expressed on cell surface membranes, or is diffused into extracellular spaces upon cell injury. The extracellular HMGB1 is able to induce a signaling cascade that activates NF-κB, leading to the synthesis of proinflammatory cytokines [6,7]. Blocking the interaction between HMGB1 and its receptors has been proved to be effective in preventing the development of diabetic nephropathy (DN) [8]. Our previous study also showed that HMGB1 is activated in DN patients and in mesangial cells in response to high glucose. In addition, HMGB1 may mediate inflammation by activating toll-like receptor pathway in DN [9]. Recently, HMGB1 is reported to be a novel regulator of ferroptosis via the RAS-JNK/p38 pathway and a potential target for treatment of leukemia [10]. However, the regulatory role of HMGB1 on ferroptosis in DN remains unknown. In the present study, we investigated the function of HMGB1 on the regulation of ferroptosis, revealing a novel function of HMGB1 in ferroptosis and providing additional treatment option for DN.

## Materials and methods

### Clinical samples

Patients with type 2 diabetes mellitus (T2DM) associated with kidney dysfunction were recruited in the Second Hospital of Jilin University from Mar 2018 to November 2019. Thirty cases healthy subjects without family history of diabetes were enrolled in the hospital within the same period. The blood samples were collected from the cubital vein, and the serum was isolated from the whole blood sample post coagulation. The present study was approved by the Internal Review Board (IRB) of Second Hospital of Jilin University, and each participant signed the informed consent.

### Cell culture

Renal mesangial SV40-MES 13 cells were obtained from American Type Culture Collection (ATCC) (Rockville, MD, U.S.A.) and were cultured in low glucose (5 mM) Dulbecco’s modified Eagle’s medium (DMEM; GIBCO, Rockville, MD, U.S.A.), supplemented with 10% fetal bovine serum (FBS) (Invitrogen, Carlsbad, CA, U.S.A.). The cells were incubated at 37°C, with 5% CO_2_.

### Transfections

The siRNA HMGB1 and Scr-siRNA were purchased from GenePharma Co Ltd (Shanghai). The sequence of HMGB1 siRNA was sense 5′-UCUUGACCACAGAUCUUAATT-3′, antisense 5′-UUAAGAGCUGU GGUCAAGATT-3′. The Scr-siRNA sequence was sense 5′-UUCUCCGAACGUGUCACGUTT-3′, antisense 5′-ACGUGACACGUUCGGAGAATT-3′. Transfections were performed using the Lipofectamine 2000 (Thermo Fisher Scientific, Inc., Waltham, MA, U.S.A.) according to the manufacturer’s instructions.

### Cell viability

Cell Counting Kit-8 (CCK-8) assay was performed for determining cell viability, using the CCK-8 assay kit (CCK-8, Dojindo Molecular Technologies, Kumamoto, Japan) according to the manufacturer’s protocol. In brief, cells were seeded into 96-well plates. The cell viability in each well was determined by adding 10 µl of CCK-8 solution. After further incubation at 37°C for 2 h, absorbance was measured using an enzyme-linked immunosorbent assay (ELISA) reader at a wavelength of 450 nm.

### Real-time PCR

RNA was isolated from cultured cells using the RNeasy mini-kit (Qiagen, Germany). The quantity and quality of total RNA samples was checked by Bioanalyzer RNA 6000 Nano assay (Agilent, Waldbronn, Germany). Total RNA (1 µg) was reverse transcribed into cDNA with the PrimeScript RT Master Mix Perfect Real Time (TaKaRa). Real time PCR conditions were 25–30 cycles at 95°C for 30 s, 56°C for 30 s, and 72°C for 1 min. The primers were as follows: GAPDH (forward primer): 5′-CTGGGCTACACTGAGCACC-3′ and (reverse primer): 5′-AAGTGGTCGTTGAGGGCAATG-3′; ACSL4 (forward primer): 5′- TGAACGTATCCCTGGACTAGG -3′ and (reverse primer): 5′-TCAGACAGTGTAAGGGGTGAA-3′; PTGS2 (forward primer): 5′-TGTGACTGTA CCCGGACTGG-3′ and (reverse primer): 5′-TGCACATTGTAAGTAGGTGGAC-3′; NOX1 (forward primer): 5′-CCTGATTCCTGTGTGTCGAAA-3′ and (reverse primer): 5′-TTGG CTTCTTCTGTAGCGTTC-3′; GPX4 (forward primer): 5′-AGTACAGGGGTTT CGTGTGC-3′ and (reverse primer): 5′-CATGCAGATCGACTAGCTGAG-3′. All reactions were performed in triplicate. The relative amounts of mRNA were calculated by using the comparative 2^(^^−ΔΔ*C*_t_^^)^ method.

### Lactate dehydrogenase activity assay

The total lactate dehydrogenase (LDH) activity in cell lysates was examined according to the manufacturer’s instructions of the LDH cytotoxicity assay kit (BioVision). Briefly, 2 × 10^5^ cells were seeded in a 24-well plate one day before assaying and all samples were analyzed in triplicate. Then cells were collected, washed, and extracted for protein to measure LDH activity. Results were normalized to the amount of total protein compared with the control cells.

### Nuclear extraction

Nuclear and cytoplasmic proteins were extracted using a nuclear and cytoplasmic protein extraction kit (#78833, Pierce Biotechnology, Rockford, IL, U.S.A.) according to the manufacturer’s instructions. To be brief, 2 × 10^5^ cells were harvested with trypsin-EDTA, centrifuged at 500×***g*** for 5 min, and re-suspended with PBS. After a second centrifugation at 500×***g*** for 2 min, the supernatant was discarded. The cell pellet was incubated with ice-cold CER I and then with CER II for 1 min. After centrifugation at 16000×***g***, the supernatant (cytoplasmic extract) and insoluble (nuclear extract) fractions were harvested for subsequent experiments.

### Immunofluorescence

The mesangial cells were seeded into six-well plates, fixed with 4% paraformaldehyde, and permeabilized with 0.2% Triton X-100 solution. Cells were then incubated with the anti-HMGB1 (Cell Signaling Technology, Beverly, Massachusetts, U.S.A.) at 4°C overnight. On the next day, cells were incubated with the Cy3-labeled secondary antibody (Cell Signaling Technology, Beverly, Massachusetts, U.S.A.) for 1 h at room temperature. Cells were then observed and images recorded under a fluorescence microscope (Olympus, Tokyo, Japan).

### ROS production

Serum samples or cells were incubated with chloro-methyl-dichlorofluorescein diacetate (CM-DCFDA, 10 mM) (Molecular Probes, Eugene, OR, U.S.A.). Samples were then centrifuged at 1000×***g*** for 3 min, and the pellets were resuspended in 500 µl phosphate-buffered saline (PBS). Measurements were performed on an FACSCalibur (BD Biosciences, San Jose, CA) flow cytometer.

### Malonaldehyde production

Serum malonaldehyde (MDA) levels were measured using thiobarbituric acid-reactive substance (TBARS) method. Each sample was placed in a 96-well plate and read at 535 nm in a microplate spectrophotometer reader (Benchmark Plus, Bio-Rad, Hercules, CA, U.S.A.). The serum concentration of MDA was expressed in nmol/ml.

### Western blot

Whole cells were lysed in 1× SDS sample buffer and the concentration of protein sample was measured by the BCA method (Beyotime, Shanghai, China). Then, 20 µg samples were separated using 10% SDS/PAGE and transferred to nitrocellulose membranes. After blocking in 5% skim milk at room temperature for 1 h, the membranes were probed with primary antibodies against HMGB1 (1:1000, #6893; Cell Signaling Technology, Beverly, Massachussets, U.S.A.), TLR3 (1:1000, #14358 Cell Signaling Technology, Beverly, Massachussets, U.S.A.), NF-κB p65 (1:1000, #8242 Cell Signaling Technology, Beverly, Massachussets, U.S.A.), phospho-NF-κB p65 (1:1000, #3033 Cell Signaling Technology, Beverly, Massachussets, U.S.A.), and nuclear factor erythroid 2-related factor 2 (Nrf2; 1:1000, #12721 Cell Signaling Technology, Beverly, Massachussets, U.S.A.) at 4°C overnight, and then incubated with appropriate horseradish peroxides-conjugated secondary antibodies for 1 h followed by detection with a Super Signal Enhanced Chemiluminescence kit (Pierce, Rockford, IL). For sequential blotting, the membranes were stripped with Stripping Buffer (Pierce) and re-probed with proper antibodies.

### Statistical analysis

The results were calculated as the mean ± the standard derivation (SD). Significances between groups were evaluated using Student’s *t* test and one-way ANOVA. Values with *P*<0.05 were considered statistically significant.

## Results

### Elevated levels of ferroptosis in DN patients

Firstly, we detected the levels of ferroptosis in patients with DN and healthy controls. There were 14 males and 16 females with an average age of 55.07 ± 4.14 years in the DN group; and 10 males and 20 females were included in the control group with well-matched age of 52.53 ± 5.17 (*P*=0.133). The detailed values of body-mass index (BMI), fasting plasma glucose (FPG), glycated hemoglobin A1c (HbA1c), total cholesterol (TC), triglyceride (TG), high-density lipoprotein cholesterol (HDL-C), low-density lipoprotein cholesterol (LDL-C), fasting insulin (FINS), homeostasis model assessment of insulin resistance (HOMA-IR) etc. in the DN or control group were shown in Table 1. Not surprisingly, patients with DN had significant higher levels in terms of FPG, HbA1c, TG, TC, BUN etc (Table 1).

**Table 1:** General clinical characteristics in DN patients and healthy participants

	NC (*n*=30)	DN (*n*=30)	*P*
Sex			
Male	10 (33.3%)	14 (46.7%)	0.191
Female	20 (66.7%)	16 (53.3%)	
Age (year)	52.53 ± 5.17	55.07 ± 4.14	0.133
BMI (kg.m^−2^)	23.93 ± 1.11	24.43 ± 0.86	0.389
Course of disease	/	3.51 ±1.54	0.113
FPG (mmol/l)	4.83 ± 0.69	10.88 ± 1.55	<0.001
HbA1c (%)	5.24 ± 0.35	10.25 ± 0.98	<0.001
FINS (mU/l)	8.63 ± 1.29	23.20 ± 5.90	<0.001
HOMA-IR	2.10 ± 0.36	15.35 ± 3.50	<0.001
Cr (µmol/l)	53.90 ± 4.57	85.00 ± 18.18	<0.001
TG (mmol/l)	0.78 ± 0.24	4.94 ± 0.89	<0.001
TC (mmol/l)	2.79 ± 0.44	6.44 ± 1.63	<0.001
HLD-C (mmol/l)	1.10 ± 0.13	1.15 ± 0.19	0.264
LDL-C (mmol/l)	2.88 ± 0.12	2.63 ± 0.65	0.115
BUN (mmol/l)	4.87 ± 1.35	12.90 ± 1.20	<0.001
ACR (mg/g)	7.18 ± 4.12	36.29 ±3.64	<0.001
eGFR (ml/min/1.73 m^2^)	130.13 ± 12.62	70.53 ± 10.18	<0.001

Abbreviation: ACR, creatinine ratio; BMI, body-mass index; BUN, blood urea nitrogen; Cr, creatinine; eGFR, estimated GFR; FINS fasting insulin; FPG, fasting plasma glucose; HbA1c, glycated hemoglobin A1c; HDL-C, high-density lipoprotein cholesterol; HOMA-IR, homeostasis model assessment of insulin resistance; LDL-C, low-density lipoprotein cholesterol; TC, total cholesterol; TG, triglyceride.

**Figure 1:**
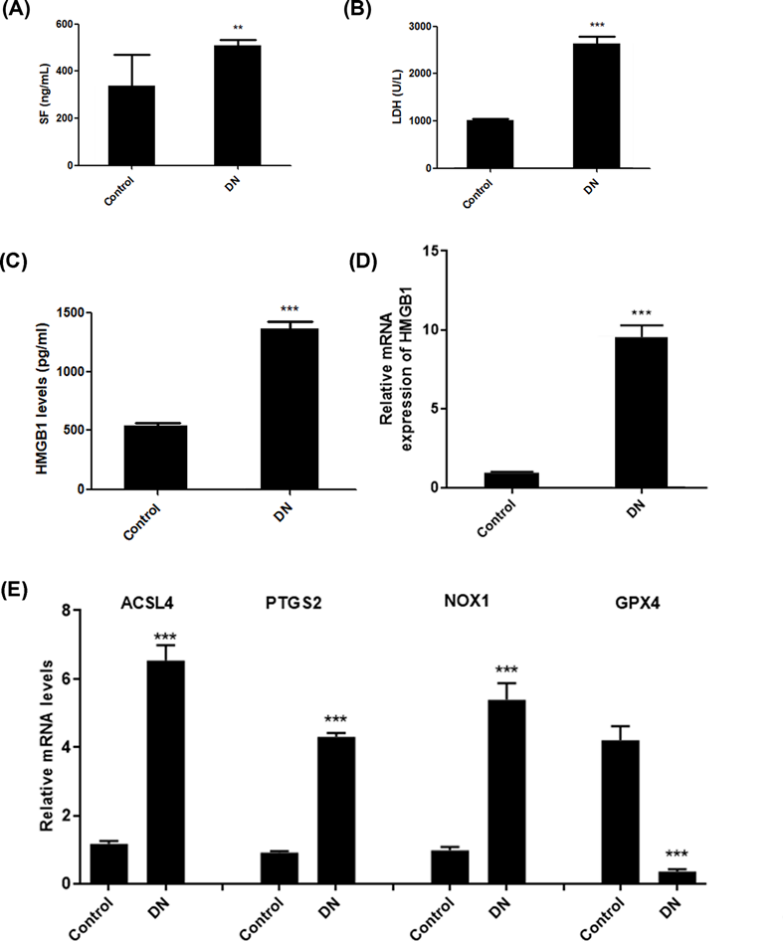
Elevated levels of ferroptosis in DN patients Levels of serum ferritin (**A**) and LDH (**B**) in DN patients and healthy controls. Levels of serum HMGB1 (**C**) and mRNA expression of HMGB1 (**D**) in DN patients and healthy controls. Real-time PCR was used to detect the expression of ferroptosis-related proteins, including ACSL4, PTGS2, NOX1, and GPX4 (**E**). **P<0.01, ***P<0.001, compared with control.

The indicators for ferroptosis included release of serum ferritin and LDH, and production of ROS and MDA. Consequently, we observed up-regulated release of serum ferritin (Figure 1A) and LDH (Figure 1B) in DN patients. Moreover, HMGB1 was also markedly up-regulated in the serum of DN patients (Figure 1C,D). In addition, real-time PCR was used to detect the expression of ferroptosis-related proteins, including ACSL4, PTGS2, NOX1, and GPX4. Data showed that patients with DN exhibited elevated expression of ACSL4, PTGS2, and NOX1, and decreased GPX4 levels (Figure 1E). These data suggest that ferroptosis levels are enhanced in the pathogenesis of DN.

### High glucose induced ferroptosis in mesangial cells

**Figure 2:**
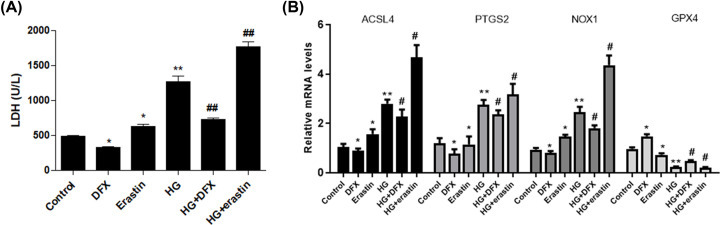
High glucose induced ferroptosis in mesangial cells SV40 MES 13 cells were cultured and treated with eratin and DFX alone or combined with high glucose. Production of LDH was detected in each group (**A**). Real-time PCR was performed to determine levels of ACSL4, PTGS2, NOX1, and GPX4 (**B**). *P<0.05, **P<0.01, compared with control. ^#^*P*<0.05, ^##^*P*<0.01, compared with HG group.

Erastin, a classic inducer of ferroptosis, was used to evaluate this aspect in high-glucose (25 mM) treated mesangial cells. We measured cell membrane permeability by LDH release assay. High glucose (25 mM) and eratin (5 µM) both significantly induced LDH release, whereas iron chelation agent DFX (200 µM) reversed glucose-induced LDH release in SV40 MES 13 cells (Figure 2A). Moreover, real-time PCR showed exposure to eratin and glucose promoted expression of ACSL4, PTGS2, and NOX1, and decreased GPX4 levels; whereas DFX suppressed ACSL4, PTGS2, and NOX1, and increased GPX4 levels in glucose-treated cells (Figure 2B). Collectively, these data suggest that high glucose could induce ferroptosis in mesangial cells.

### HMGB1 mediates the glucose-induced ferroptosis in mesangial cells

**Figure 3:**
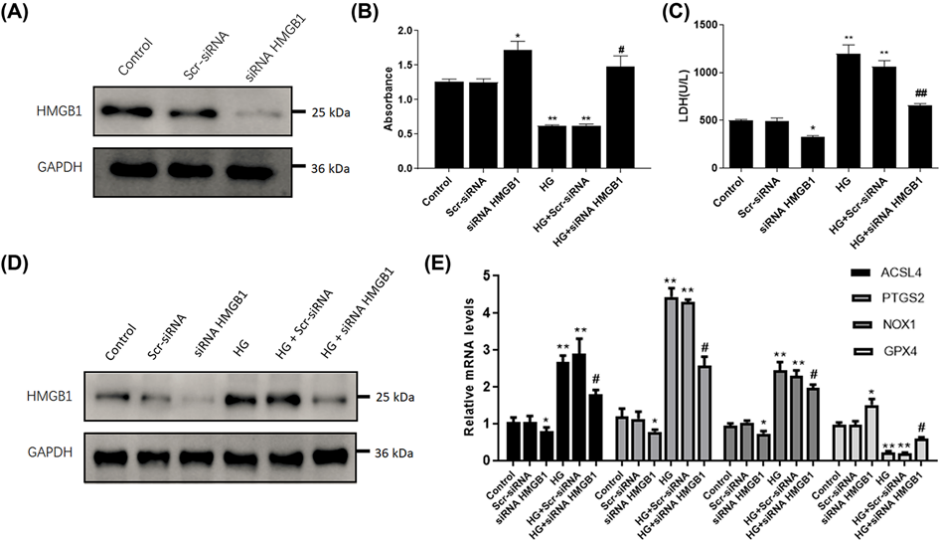
HMGB1 mediates the glucose-induced ferroptosis in mesangial cells SV40 MES 13 cells were cultured and transfected with HMGB1 siRNA or scramble siRNA (Scr-siRNA), followed by high glucose (25 mM) treatment. (**A**) Determination of protein levels of HMGB1 by Western blot. (**B**) Cell viability in each group was determined with CCK-8 assay. (**C**) ELISA was used to detect the production of LDH. (**D**) Extracellular HMGB1 protein levels were determined by Western blot. (**E**) Real-time PCR was performed to determine levels of ACSL4, PTGS2, NOX1, and GPX4. *P<0.05, **P<0.01, compared with control. ^#^*P*<0.05, ^##^*P*<0.01, compared with HG group.

To identify the role of HMGB1 in glucose-induced ferroptosis in SV40 MES 13 cells, we then manipulated the HMGB1 level in mesangial cells via transfecting HMGB1-siRNA, which was validated using Western blot (Figure 3A). As a result, CCK-8 showed that high glucose treatment dramatically decreased cell viability, whereas transfection with HMGB1 siRNA significantly elevated SV40 MES 13 cell viability (Figure 3B). To explore the role of HMGB1 on ferroptosis, we measured cell membrane permeability by the LDH release assay. High glucose significantly induced LDH release, whereas depletion of HMGB1 suppressed the release of LDH (Figure 3C). In addition, Western blot showed that transfection with HMGB1 siRNA significantly decreased the extracellular levels of HMBG1 in SV40 MES 13 cells with or without glucose treatment (Figure 3D). Furthermore, real-time PCR was used to detect the expression of ferroptosis-related biomarkers. We found that glucose-treated SV40 MES 13 cells exhibited elevated expression of ACSL4, PTGS2, and NOX1, and decreased GPX4 levels; whereas depletion of HMGB1 suppressed ACSL4, PTGS2, and NOX1, and increased GPX4 levels (Figure 3E). Collectively, these data revealed the suppression of HMGB1 prevents against glucose-induced ferroptosis in mesangial cells.

### HMGB1 regulates ferroptosis-induced oxidative stress upon exposure to high glucose

**Figure 4:**
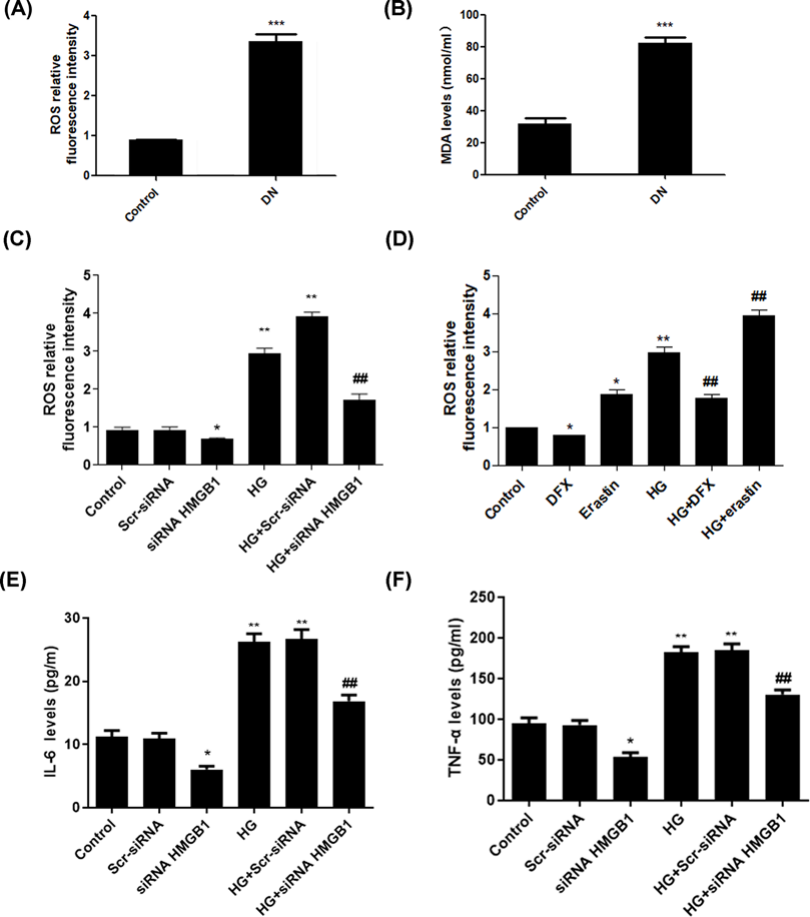
HMGB1 regulates ferroptosis-induced oxidative stress upon exposure to high glucose Levels of ROS (**A**) and MDA (**B**) in DN patients and healthy controls. SV40 MES 13 cells were transfected with HMGB1 siRNA and stimulated with high glucose. The production of ROS (**C**), IL-6 (**E**) and TNF-α (**F**) was detected. Furthermore, SV40 MES 13 cells were treated with eratin and DFX alone or combined with high glucose, and the levels of ROS were detected (**D**). *P<0.05, **P<0.01, ***P<0.001, compared with control. ^##^*P*<0.01, compared with HG group.

Ferroptosis is characterized by excessive oxidative stress leading to lipid peroxidation and the rupture of membrane permeability. The redox-sensitive probe CM-DCFDA, whose fluorescence reflects the production of ROS, was used to evaluate this aspect. We observed up-regulated production of ROS and MDA in the serum of DN patients (Figure 4A,B). In addition, data showed that high glucose induced extensive oxidative stress that was prevented by knockdown of HMGB1 (Figure 4C). To validate the role of ferroptosis in such oxidative status, we applied iron chelation with DFX and erastin, a classic inducer of ferroptosis. Data showed that extensive oxidative stress was prevented by DFX with or without exposure to high glucose. Whereas, the production of ROS was even elevated upon treatment with ferroptosis inducer erastin (Figure 4D). In order to explore the role of HMGB1 in inflammatory response, we detected inflammatory cytokines upon exposure to high glucose. We found that glucose-treated SV40 MES 13 cells exhibited elevated production of IL-6 and TNF-α, which were suppressed by interference of HMGB1 in SV40 MES 13 cells (Figure 4E,F). Collectively, these data suggest that HMGB1 regulates the ferroptosis-induced oxidative stress upon exposure to high glucose.

### HMGB1 regulates glucose-induced ferroptosis via Nrf2 signaling pathway

**Figure 5:**
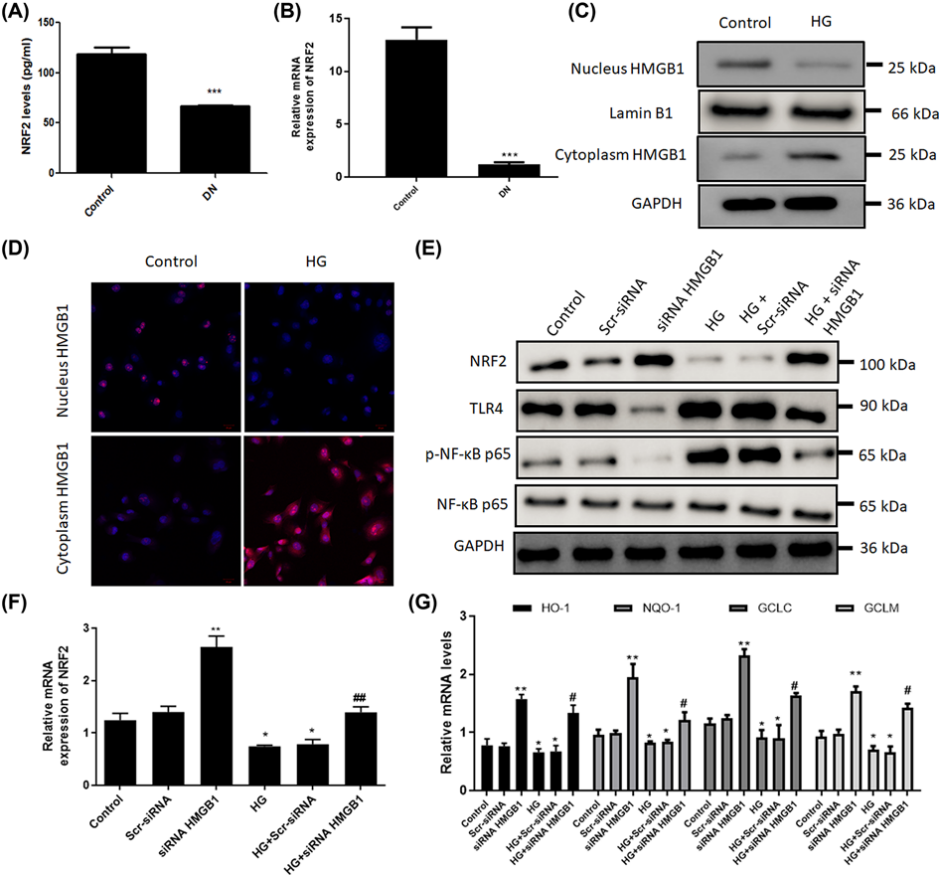
HMGB1 regulates glucose-induced ferroptosis via Nrf2 signaling pathway ELISA and real-time PCR assays were performed to determine levels of Nrf2 in DN patients and healthy controls (**A,B**). SV40 MES 13 cells were transfected with HMGB1 siRNA or scramble siRNA and stimulated with high glucose (25 mM). Western blot and immunofluorescence staining were performed to detect expression of HMGB1 in the cytoplasm and nucleus in glucose-treated SV40 MES 13 cells (**C,D**). (**E**) The protein levels of Nrf2, TLR4, and NF-κB p65 were determined using Western blot. (**F,G**) Relative mRNA levels of Nrf2 and its downstream targets, including HO-1, NQO-1, GCLC and GCLM, were detected using real-time PCR in SV40 MES 13 cells after siRNA treatment. *P<0.05, **P<0.01, ***P<0.001, compared with control. ^#^*P*<0.05, ^##^*P*<0.01, compared with HG group.

Nrf2 is a critical transcription factor for protecting against oxidative injury. ELISA and real-time PCR assays showed that the serum Nrf2 was significantly decreased in DN patients compared with control subjects (Figure 5A,B). Cellular component analysis revealed that stimulation with high glucose led to cytoplasm translocation of HMGB1 from nucleus (Figure 5C,D). Furthermore, treatment with high glucose obviously decreased the mRNA and protein levels of NRF2, whereas knockdown of HMGB1 promoted Nrf2 expression in mesangial cells (Figure 5E,F). Additionally, high glucose notably increased TLR4 and p-NF-κB p65, while HMGB1 siRNA suppressed high glucose-induced activation of TLR4/NF-κB signaling pathway in mesangial cells (Figure 5E). Moreover, suppression of HMGB1 reversed glucose-induced reduction in Nrf2 downstream targets, including HO-1, NQO-1, GCLC and GCLM, in SV40 MES 13 cells (Figure 5G). Taken together, these findings suggest that HMGB1 regulates glucose-induced ferroptosis via Nrf2 signals in mesangial cells.

## Discussion

HMGB1 is a DNA-binding nonhistone protein and implicated in DNA replication, transcription, and repair. In addition to its nuclear functions, extracellular HMGB1 is considered to be a proinflammatory mediator in human diseases [11]. In the present study, elevated levels of HMGB1 were observed in DN patients accompanied with deregulated ferroptosis-associated markers. Furthermore, our results identified a regulatory role of HMGB1 in ferroptosis of mesangial cells, suggesting a novel therapeutic target for DN.

Since the concept of ferroptosis was first reported, accumulating studies have revealed that this novel form of cell death is closely implicated in the pathogenesis and progression of human diseases, such as cancer, arteriosclerosis, and neurodegenerative diseases. Ferroptosis is a form of iron-dependent, non-apoptotic regulated cell death, characterized by the accumulation of lethal lipid hydroperoxides and loss of the activity of the lipid repair enzyme [12]. Recent studies have demonstrated that ferroptosis have implications in diverse kidney diseases, such as polycystic kidney disease, acute kidney injury, and renal cell carcinoma. In addition, it is found that ferroptosis is involved in diabetes myocardial ischemia/reperfusion injury, islet viability and function, and diabetic cardiomyopathy [13–15]. However, it is still unclear the regulatory role of ferroptosis in diabetic kidney disease. In the present study, we observed up-regulated levels of ferritin, LDH, ROS, and MAD, indicators for ferroptosis, in DN patients. It has been suggested that induction of ferroptosis is associated with elevation of ACSL4, PTGS2, and NOX1, and reduced GPX4 [16]. Strikingly, patients with DN exhibited elevated ACSL4, PTGS2, and NOX1, and decreased GPX4 levels. Consistently, *in vitro* assay confirmed that high glucose and eratin, a classic inducer of ferroptosis [17], both induced ferroptosis, which was reversed by treatment with iron chelation DFX in SV40 MES 13 cells. Collectively, these findings primarily reveal that high glucose could confer to ferroptosis in mesangial cells in response to high glucose.

In the context of DN, up-regulation of HMGB1 expression is detected in the renal tubules of human kidneys with DN. Stimulation with high glucose promotes release of endogenous HMGB1 by tubular epithelial cells and podocytes and blockade of HMGB1 attenuates diabetic kidney injury *in vivo* [18,19]. We also found that high glucose promotes translocation of endogenous HMGB1 to cytoplasm. In order to explore the regulatory role of HMGB1 in ferroptosis, we manipulated the HMGB1 level in mesangial cells. Consequently, suppression of HMGB1 restored cellular proliferation, prevented ROS production, reversed ferroptosis, and decreased proinflammatory cytokines in mesangial cells exposed to high glucose. These findings demonstrated that suppression of HMGB1 confers to a beneficial role against glucose-induced ferroptosis, excessive oxidation, and inflammation in mesangial cells.

Nrf2 is a transcription factor which was identified as a master regulator of defensive responses to oxidative stress [20]. Under external stimuli, Nrf2 will be deubiquitinated, translocated to the nucleus and interacted with the antioxidant response element (ARE) to activate the transcription of its target genes. As oxidative stress and inflammatory response are important mediator of progression of diabetic kidney disease, it is reasonable to develop Nrf2 as a novel target for this disease [21,22]. One of the recent findings of Nrf2 signaling pathway is against ferroptosis [23,24]. Additionally, the HMGB1/TLR4/NF-κB and Nrf2/HO1 pathways have been involved in the cisplatin-induced nephrotoxicity [25,26]. In the present study, we explored whether HMGB1 regulates ferroptosis dependent on Nrf2 signaling in the context of diabetic kidney disease. Clinical samples with DN and high glucose-treated mesangial cells exhibited decreased Nrf2 as well as its downstream targets including HO-1, NQO-1, GCLC, and GCLM. Moreover, suppression of HMGB1 reversed glucose-induced activation of TLR4/NF-κB and reduction in Nrf2 as well as its targets in SV40 MES 13 cells, suggesting HMGB1 is a novel regulatory of ferroptosis via Nrf2 is implicated in HMGB1 regulates glucose-induced signals in mesangial cells.

In conclusion, the present study suggests that HMGB1 is translocated from the nucleus to the cytoplasm in high glucose-treated mesangial cells and serves as a positive regulator of ferroptosis dependent on Nrf2 signaling pathway. These findings provide novel therapeutic strategies targeting HMGB1 and ferroptosis in diabetic kidney disease.

## Data Availability

The datasets generated and/or analyzed during the current study are available from the corresponding author on reasonable request.

## Abbreviations

CM-DCFDA: chloro-methyl-dichlorofluorescein diacetate
DN: diabetic nephropathy
ESRD: end-stage renal disease
HMGB1: high-mobility group box-1
LDH: lactate dehydrogenase
MDA: malonaldehyde
Nrf2: nuclear factor erythroid 2-related factor 2
PBS: phosphate-buffered saline
ROS: reactive oxygen species
